# GC‐MS metabolomics‐based approach for the identification of a potential VOC‐biomarker panel in the urine of renal cell carcinoma patients

**DOI:** 10.1111/jcmm.13132

**Published:** 2017-04-04

**Authors:** Márcia Monteiro, Nathalie Moreira, Joana Pinto, Ana S. Pires‐Luís, Rui Henrique, Carmen Jerónimo, Maria de Lourdes Bastos, Ana M. Gil, Márcia Carvalho, Paula Guedes de Pinho

**Affiliations:** ^1^ UCIBIO@REQUIMTE Laboratory of Toxicology Faculty of Pharmacy University of Porto Porto Portugal; ^2^ Cancer Biology & Epigenetics Group Portuguese Oncology Institute of Porto (IPOPorto) Porto Portugal; ^3^ Department of Pathology Portuguese Oncology Institute of Porto (IPOPorto) Porto Portugal; ^4^ Department of Pathology and Molecular Immunology‐Biomedical Sciences Institute (ICBAS) University of Porto Porto Portugal; ^5^ CICECO‐Instituto de Materiais de Aveiro (CICECO/UA) Departamento de Química Universidade de Aveiro Aveiro Portugal; ^6^ UFP Energy Environment and Health Research Unit (FP‐ENAS) Universidade Fernando Pessoa Porto Portugal

**Keywords:** volatile organic compounds, urine, biomarkers, renal cell carcinoma, gas chromatography–mass spectrometry, multivariate statistical analysis

## Abstract

The analysis of volatile organic compounds (VOCs) emanating from biological samples appears as one of the most promising approaches in metabolomics for the study of diseases, namely cancer. In fact, it offers advantages, such as non‐invasiveness and robustness for high‐throughput applications. The purpose of this work was to study the urinary volatile metabolic profile of patients with renal cell carcinoma (RCC) (*n* = 30) and controls (*n* = 37) with the aim of identifying a potential specific urinary volatile pattern as a non‐invasive strategy to detect RCC. Moreover, the effect of some confounding factors such as age, gender, smoking habits and body mass index was evaluated as well as the ability of urinary VOCs to discriminate RCC subtypes and stages. A headspace solid‐phase microextraction/gas chromatography–mass spectrometry‐based method was performed, followed by multivariate data analysis. A variable selection method was applied to reduce the impact of potential redundant and noisy chromatographic variables, and all models were validated by Monte Carlo cross‐validation and permutation tests. Regarding the effect of RCC on the urine VOCs composition, a panel of 21 VOCs descriptive of RCC was defined, capable of discriminating RCC patients from controls in principal component analysis. Discriminant VOCs were further individually validated in two independent samples sets (nine RCC patients and 12 controls, seven RCC patients with diabetes mellitus type 2) by univariate statistical analysis. Two VOCs were found consistently and significantly altered between RCC and controls (2‐oxopropanal and, according to identification using NIST14, 2,5,8‐trimethyl‐1,2,3,4‐tetrahydronaphthalene‐1‐ol), strongly suggesting enhanced potential as RCC biomarkers. Gender, smoking habits and body mass index showed negligible and age‐only minimal effects on the urinary VOCs, compared to the deviations resultant from the disease. Moreover, in this cohort, the urinary volatilome did not show ability to discriminate RCC stages and histological subtypes. The results validated the value of urinary volatilome for the detection of RCC and advanced with the identification of potential RCC urinary biomarkers.

## Introduction

RCC, the most common and lethal malignancy of the kidney 1, is traditionally detected by classical imaging techniques, such as ultrasound, computed tomography and magnetic resonance imaging 1, 2. However, most RCCs lack characteristic early clinical symptoms and persist asymptomatic until later stages 2, 3, when the response to therapy is limited and the prognosis poorer with a dismal possibility of cure 4. In view of these aspects, the markedly importance of investigating new diagnostic assays that allow early detection and diagnosis and the clinical impact that they could have in the clinical management of RCC is highlighted 5, 6.

Throughout the past decade, the considerable refinement of new analytical techniques, such as mass spectrometry (MS), usually coupled to separation techniques (namely, gas chromatography, GC) and nuclear magnetic resonance (NMR) spectroscopy, has made it possible to more easily detect and identify compounds in complex mixtures, providing a way to perform metabolic profiling of body fluids 7, 8, 9, 10. The exhaustive exploration of metabolomics potentialities for biomarker discovery, particularly regarding cancer, is linked to the opportunity that it offers to follow, most of the times non‐invasively, metabolic alterations accompanying the pathology. From this perspective, following distinctive deviations in a biofluid such as urine seems even more advantageous regarding its non‐invasiveness and easy collection.

Since ancient times that physicians have been correlating specific odours to specific ailments, as reviewed by several authors 11, 12, 13, and it is assumed that pathological processes may influence the individual odour fingerprint by changing the type or the relative amount of VOCs that are usually produced 11. Regarding this, the analysis of the volatile profile (volatilome), through the analysis of VOCs, is one of the most promising metabolomics‐derived approaches and, in fact, different volatile patterns have been correlated with a plethora of diseases, including cancer 11, 14, 15. Moreover, after trained dogs successfully discriminate patients with cancer from controls on the basis of urine odour 16, 17, 18, 19, 20, the potential of VOCs for the analysis of cancer was even more sustained by the development of some promising sensors array which function is dependent on the detection of VOCs emanating directly from cancer lesions 21, 22, breath 23 and urine 24. These studies provide a convincing support that the presence of cancer cells in the body leads to the alteration of VOCs emanating from biological samples, particularly of those closely correlated with tumoral tissues. The analysis of volatiles holds other advantages because sample preparation is simpler and faster, compared to other approaches, which prevents metabolite loss and enables high‐throughput processing and analysis of a plethora of compounds from several samples 25.

This study consists of an extension of our previous work in which we developed a headspace solid‐phase microextraction (HS‐SPME) gas chromatography–mass spectrometry (GC‐MS) methodology to perform the volatile profiling of human urine samples as a way to detect changes characteristics of RCC. In fact, our previous work 26 demonstrated, in a pilot study, the potential of the urinary volatilome to successfully segregate RCC patients and controls after multivariate statistical analysis. Here, we tested the analytical protocol using a larger cohort. Besides the effect of the disease over the urinary volatilome, the effect of the following possible confounding factors body mass index (BMI), age, gender and smoking habits was also evaluated, and the ability of urinary VOCs to discriminate samples according to RCC histological subtypes and stages was assessed. Moreover, we used two independent sets of samples, including controls and RCC with and without diabetes mellitus type 2 (DMT2), to validate the 21 compounds that, according to univariate statistical analysis, showed to be descriptive of RCC. DMT2 is a relatively common comorbidity in patients with RCC and has been proposed as a possible risk factor for RCC's development 27; however, it is known to independently produce alterations on the metabolic profile, namely on the urinary volatile profile 28, 29, 30. Thus, the validation of any discriminant compounds in patients with DMT2 would be of great importance to assess the robustness of potential RCC biomarkers.

## Material and Methods

### Chemicals

The chemicals used during the experimental work were all of analytical grade. Sodium chloride (NaCl, 99.5%) and hydrochloric acid (HCl, 37%) were purchased from Sigma‐Aldrich (St. Louis, MO, USA). The water used to prepare the 6M solution of HCl was ultrapure water obtained from a Milli‐Q system (Millipore, Bedford, MA, USA).

### Subjects

The Portuguese Oncology Institute‐Porto (IPO‐Porto) cordially provided the urine samples from patients diagnosed with RCC and samples from control subjects (cancer‐free) were kindly provided by the Cedofeita Clinical Analysis Laboratory (Porto, Portugal). All subjects enrolled in the study gave written informed consent, and the study was approved by the Ethics Committee of IPO‐Porto (CES76/2012).

For the construction of the classification model, the cohort enrolled in this study comprised 30 patients diagnosed with primary RCC (11 females and 19 males; age range 35–79, average age 60) and 37 healthy control (cancer‐free) subjects (27 females and 10 males; age range 38–86, average age 69; Table 1). Subjects with type II diabetes mellitus and other acute pathological conditions were left out from the study. Table 1 includes the classification of the RCC tumours regarding the histopathological subtype and TNM staging 2. For RCC patients, information on subject age, gender, smoking habits and BMI is also displayed on the table. For controls, only description on age and gender was available.

**Table 1:** List of urine samples collected for controls and RCC subjects, comprising number of samples, age and gender

Sample group	n^o^. samples	Age range	Mean age ± S.D.	Females	Males
Controls (total cohort)	37	50–86	69.08 ± 12.04	27	10
RCC (total cohort)	30	35–79	59.95 ± 12.44	11	19
Clear‐ cell (ccRCC)	20	41–79	61.47 ± 12.24	5	15
Type 1 papillary (pRCC)	5	24–74	61.53 ± 7.34	3	2
Chromophobe (chRCC)	5	35–71	52.28 ± 16.34	3	2
With metastases	8	50–78	66.05 ± 8.62	1	7
Without metastases	22	35–79	57.73 ± 13.02	10	12
Stage I	16	41–79	58.50 ± 12.13	8	8
Stage II	3	42–78	59.99 ± 17.91	3	3
Stage III	7	35–76	61.74 ± 14.70	2	5
Stage IV	4	50–72	62.58 ± 9.20	‐	4
Smokers a ^,^ b	9	35–76	55.20 ± 13.16	2	7
Non‐smokers	21	41–79	62.86 ± 11.00	10	11
BMI ≥25^c^	20	35–79	61.02 ± 11.80	9	11
BMI <25	7	42–77	60.84 ± 14.45	1	6
Controls >60 year *versus* Controls ≤60 year, gender‐matched					
Age *≤*60	12	50–60	54.75 ± 4.05	8	4
Age >60	13	67–86	80.62 ± 5.84	9	4
ccRCC *versus* other RCC subtypes, age‐matched					
ccRCC	9	41–74	57.81 ± 12.56	1	8
Other subtypes	9	35–74	56.79 ± 13.68	5	4

For RCC patients, histopathological cancer type, TNM staging, presence or absence of metastases, smoking habits and BMI (kg.m‐2).

S.D., standard deviation.

a Information not available for 1 subject.

b Includes smokers (*n* = 4) and former smokers (*n* = 5).

c Information not available for three subjects.

The urine samples from all the patients were provided before surgery, radiation and/or implementation of any chemotherapeutic schedules. Both patients and controls provided a sample of first void urine (after overnight fasting) in a sterile cup. All samples were then centrifuged (2916 × g for 20 min. at 4°C) and split into several aliquots. The aliquots of urine samples were stored into cryovials at −80°C until further analysis.

### Sample preparation

Prior to HS‐SPME/GC‐MS, urine samples were thawed (room temperature), 2 ml of urine was acidified (pH 2.00 ± 0.02; adjusted with 6M hydrogen chloride and transferred to a 10‐ml glass vial containing 0.59 g of sodium chloride, capped with a PTFE septum and a screw cap). Each sample was tested in duplicate.

### HS‐SPME/GC‐MS measurements

The HS‐SPME procedures were performed using a Combi‐PAL autosampler (Varian Pal Autosampler, Switzerland) and the Cycle Composer software (CTC Analytics System Software, Switzerland) using a DVB/PDMS fibre coating, as optimized in a previous work 26.

The GC‐IT/MS analyses of the volatiles extracted from urine were performed using a Varian CP‐3800 gas chromatograph equipped with a Varian Saturn 4000 ion trap mass detector and a Saturn GC‐IT/MS workstation software (version 6.8). Chromatographic separation was carried out using a capillary column VF‐5 ms (30 m × 0.25 mm× 0.25 μm) from Varian. High purity helium C‐60 (Gasin, Portugal) was used as the carrier gas at a constant flow rate of 1.0 ml/min. The oven temperature was held for 1 min. at 40°C and then increased at a rate of 5°C/min to 250°C (held for 5 min.) followed by an increase in 5°C/min to 300°C (held for 1 min.). The detection was performed using an ion trap detector set as follows: the transfer line, manifold and trap temperatures were 280, 50 and 180°C, respectively. The mass range was 40–400 m/z, with a scan rate of 6 scans. The emission current was 50 μA, and the electron multiplier was set in relative mode to autotune procedure. The maximum ionization time was 25,000 μs, with an ionization storage level of 35 m/z. The analysis was performed in full scan mode.

The tentative assignment of the volatiles was based on the comparison of their retention times (RTs), Kovats retention index (RIs) and mass spectra to those from the National Institute of Standards (NIST) mass spectral library (2014). When possible, the identification was confirmed with the injection of available standards using the same column and temperature programme. Only for forward and reverse % of match of 70% or above the tentative compound identification was considered. The integration of the selected variables was performed using selected qualifier ions based on their relative abundance and selectivity. The RI of these variables was calculated according to the RTs obtained, under the same chromatographic conditions, for a solution of *n‐*alkanes (C8‐C20) series (Table S1A). Unidentified compounds are reported as ‘VOC_*i*_’ (*i *=* *1,2,3,…), throughout the text, according to their crescent RT value.

In addition to other adequate references, the Human Metabolome Database (HMDB, www.hmdb.ca) and Urine Metabolome Database (www.urinemetabolome.ca) were consulted to assist in the identification and biochemical interpretation of compounds.

### Multivariate statistical analysis

All the raw data files were exported as ASCII files and imported into Excel for manual chromatogram alignment. The MS spectra were used to confirm peak identity as a way of supervising the manual alignment. The goal of this procedure was the correction of small differences in RTs across the samples due to analytical drifts. Manual alignment was preferred, despite being a time‐consuming process. The matrix corresponding to the sample with higher number of scans (expressed as kcounts) was used as reference for the manual alignment, and all the other samples were aligned against it. As all the samples were analysed as duplicates, a mean chromatogram was obtained for each. After alignment, the chromatographic region between 1.31 and 44.00 min. was considered for multivariate analysis, after exclusion of a chromatographic region with a chromatographic signal with an expressive tailing (4.67–5.90 min.) (MATLAB 7.12.0, The MathWorks, Inc, Natick, MA, USA). Chromatograms were normalized by probabilistic quotient normalization (PQN) (Matlab 7.12.0, The MathWorks, Inc) and scaled to unit variance (UV) (SIMCA‐P 11.5, Umetrics, Umea, Sweden). Principal component analysis (PCA) and partial least‐squares discriminant analysis (PLS‐DA) were applied to the chromatograms (SIMCA‐P 11.5, Umetrics, Umea, Sweden). PLS‐DA model robustness was assessed by Monte Carlo cross‐validation (MCCV) using 500 iterations, using a software developed in the University of Aveiro 31. For each generated classification models, *Q* ^2^ values, number of latent variables and confusion matrices of original and randomly permuted classes were retrieved. Sensitivity (sens), specificity (spec) and classification rates (CR) were computed. A receiver operating characteristics (ROC) map was constructed to assess the predictive power of each model. PLS‐DA models were considered robust when only minimal overlap of the original and randomly permuted *Q* ^2^ distributions was observed.

A variable selection method was applied to the aligned and normalized matrices in order to increase model robustness and decrease the impact of potentially noisy variables. The chromatographic variables were selected through the intersection of three conditions: VIP > 1 and VIP/VIP_cvSE _> 1 and |b/b_cvSE_|  > 1 32. After the application of variable selection, each PLS‐DA was reapplied and resubmitted to MCCV. For each model, the chromatographic features (characterized by RT and mass spectra) found to contribute to class discrimination were integrated in the original chromatograms (Saturn GC‐IT/MS workstation version 6.8). The average value for each feature was obtained and PQN‐normalized (Matlab 7.12.0, The MathWorks, Inc). The statistical significance of each normalized integral was evaluated by the nonparametric Wilcoxon rank‐sum test or the parametric two‐sample Student's *t*‐test. Chromatographic features were considered statistically relevant when *P* value <0.05. The Benjamini–Hochberg false discovery rate (BH‐FDR) correction 33 was applied to adjust *P*‐values for multiple comparisons. The FDR‐corrected *P*‐value is equal to *P‐*value*(*n*/ (n−2)), where *n* is the number of chromatographic features tested in univariate statistical analysis. Similarly, the cut‐off value considered to discriminate the statistically significant features was 0.05. Moreover, for each feature integrated, the effect size (following the definition given in Berben *et al*. 34) and percentage of variation and uncertainty were calculated. Finally, a correlation network was computed using the set of significantly different VOCs in RCC patients compared with controls, based on Spearman's rank correlation. The correlation network was computed using the Gephi 0.9.1 software (The Gephi Consortium, Paris, France) 35, considering pairs with a correlation coefficient (*r*) and a significance (*P*) threshold of |*r*| ≥ 0.7 and *P *<* *0.05.

### Validation of the descriptive VOCs

The RCC‐distinctive panel of volatiles was tested in two independent validating sets: the first consisting of nine patients diagnosed with primary RCC (six females and three males; age range 44–72, average age 60) and 12 control subjects (seven females and five males; age range 38–83, average age 59); the second consisting of seven patients diagnosed with primary RCC and with DMT2 (two females and five males; age range 40–82, average range 66; Table S1).

## Results

A total of 181 metabolites (*full scan)* were consistently detected in the urine samples of both RCC patients and healthy controls, and it was possible to identify among them several chemical classes, such as alcohols, aldehydes, ketones, terpenes, compounds containing sulphur and furan moieties, steroids and naphthalene derivatives. A representative urine full scan chromatogram with some of the most abundant VOCs is presented as supplementary material as well as a list of the selected VOCs identified for the computed PLS‐DA models (Fig. S1, Table S2).

To study the effect of the possible confounding factors, the controls were considered to study the effects of age and gender. According to the results of the MCCV (Table S3), and considering these cohort of samples used on the untargeted approach, only age showed some predictive ability to classify the samples and thus act as a confounder. In fact, when considering the age (controls ≤60 years *versus* controls >60 years) as the discriminant factor, from the selected variables of the gender‐matched cohort (Table S4), nine were found statistically significantly altered (after BH‐FDR correction) between controls over 60 years compared to controls under 60 years of age. Six of these compounds were found elevated (1,2,3,4‐tetrahydro‐1,5,7‐trimethylnaphthalene, 1,1,6‐trimethyl‐1,2‐dihydronaphthalene, 1‐(2,3,6‐trimethylphenyl)‐3‐buten‐2‐one, 4‐(2,6,6‐trimethyl‐1‐cyclohexa‐1,3‐dienyl)butan‐2‐one, 1,1,4,5,6‐pentamethyl‐2,3‐dihydro‐1H‐indene, and one unidentified) and 3 decreased (2‐pentylfuran, D‐carvone, and (4Z)‐4‐(2,2‐dimethyl‐6‐methylidene‐cyclohexylidene)‐butan‐2‐ol) in older subjects.

Considering the differences among the RCC group of samples for smoking habits and BMI, unmatched and matched PLS‐DA models were obtained for these and submitted to MCCV, but all failed to show predictive ability to classify the samples which means that, considering this cohort of samples, these factors do not seem to act as confounders and impact the classification of samples according to the presence of the disease. Moreover, using the volatile profile, it was not possible to distinguish RCC samples according to their TNM staging (Table S3). Some ability to classify samples according to histological subtype (ccRCC *versus* other subtypes) was demonstrated (median *Q* ^2 ^= 0.70, sens = 94%, spec = 78.6% and CR = 86.3%; Table S3); however, when the variables were tested for their individual significance, none was found significant.

Regarding the disease model, when considering an age‐ and gender‐matched subcohort of RCC and control samples, the predictability of the PLS‐DA model did not improve compared to the unmatched cohort (Table S3), evidencing a negligible effect of both age and gender in the urinary volatilome of this set of samples. Thus, the unmatched model was the one considered for the assessment of the impact of the variables contributing for the urinary profile of RCC.

**Figure 1:**
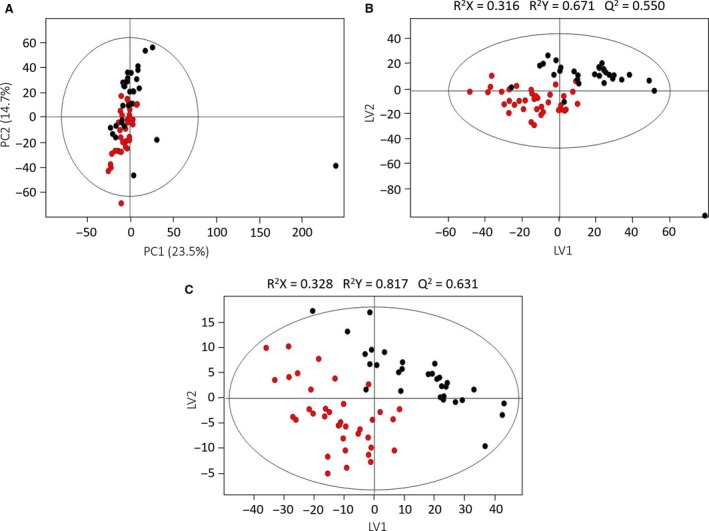
PCA (A) and PLS‐DA before (B) and after(C) variable selection scores scatter plots obtained for the HS‐SPME/GC‐MS chromatograms of human urine samples for the unmatched cohort of controls (*n* = 37, 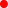) and RCC patients (*n* = 30, ●). The PCA model was obtained with 2 PCs and the PLS‐DAs with 2 LVs. The ellipses indicate the 95% confidence limit of each model.

Considering the study of the effect of RCC on the urinary volatilome, the PCA scores scatter plot showed only a slight separation trend between controls and RCC on the first principal component (PC1) but using the PLS‐DA a better discrimination was possible (R2X = 0.316; R2Y = 0.671; *Q* ^2 ^= 0.550). Moreover, the prediction power of the PLS‐DA model improved after variable selection (*Q* ^2^ = 0.631; Fig. 1) and was confirmed by MCCV (median *Q* ^2^ = 0.72, sens = 95.6%, spec = 92.6%, CR = 93.9% *versus* median *Q* ^2^ = 0.42, sens = 69.7%, spec = 81.7%, CR = 76.3, before the selection of the variables), as is expressed by the improvements in the *Q* ^2^ distribution and ROC curve (Fig. S2, Table S3). Once more, the application of a method of variables selection proved to be useful for the reduction/exclusion of redundant and irrelevant variables for the classification whereas retaining those with more predictive power.

Among the chromatographic features selected as discriminative for the PLS‐DA classification for the unmatched disease model (Fig. 1C), 2‐pentylfuran, 1,2,3,4‐tetrahydro‐1,5,7‐trimethylnaphthalene, TDN and VOC_2_ were common to the age model (Table 2) and showed possible bias of higher average age in controls than in RCC patients. Thus, the results evidence that there is only a possible small effect of the discrepancies on age on the classification of the RCC samples and, overall, considering this cohort of samples, the possible existing confounders did not hinder the classification of RCC samples. Moreover, in the unmatched disease model, from the 21 variables that showed to be significant (*P *<* *0.05) after the BH‐FDR correction, only two (VOC_2_ and 1,1,6‐trimethyl‐1,2‐dihydronaphthalene) with possible bias from the age model were present.

**Table 2:** List of VOCs extracted from urine and varying in the RCC group compared to controls, characterized by their IUPAC (and common) name, RTs and quantifier ions (*m/z*)

Metabolite	Quantifier ions (*m/z*)	RT (min)	Identification method	% variation (± % uncertainty)	ES (±ES_SE_)	*P*‐value	Corrected *P‐*value
2‐Oxopropanal (Pyruvaldehyde)	43 + 72	1.67	Std	82.96 (11.21)	1.43 (0.54)	3.90 × 10^−6^	3.99 × 10^−5^
2‐Methylpropan‐2‐ol	41 + 59 + 95	1.82	Std	71.44 (16.16)	0.88 (0.50)	1.80 × 10^−3^	3.51 × 10^−3^
2‐Ethoxy‐2‐methylpropane	41 + 59 + 87	2.17	MS	100.62 (18.92)	0.97 (0.51)	7.45 × 10^−4^	1.78 × 10^−3^
2‐Methylpropan‐1‐ol (Isobutanol)	41 + 57	2.23	Std	92.58 (28.48)	0.62 (0.49)	1.11 × 10^−2^	1.63 × 10^−2^
2‐Methylbutan‐2‐ol	43 + 59 + 73	2.34	MS	32.15 (10.63)	0.68 (0.50)	1.10 × 10^−2^	1.63 × 10^−2^
Pentane‐2‐one	43 + 58 + 71 + 86	2.74	Std	68.54 (20.79)	0.65 (0.49)	8.72 × 10^−3^	1.48 × 10^−2^
2,2,5,5‐Tetramethyltetrahydrofuran	43 + 95	3.81	MS	121.89 (18.10)	1.17 (0.52)	1.72 × 10^−2^	5.91 × 10^−4^
1‐Methyl‐1,4‐cyclohexadiene	91 + 113	3.86	MS	39.74 (25.97)	0.34 (0.49)	2.38 × 10^−1^	2.74 × 10^−1^
4‐Methylheptan‐2‐one	43 + 85 + 58	7.80	MS	85.48 (14.54)	1.14 (0.52)	1.95 × 10^−5^	6.03 × 10^−4^
Phenol	66 + 94	9.02	MS	5.02 (30.84)	0.04 (0.48)	9.30 × 10^−1^	9.30 × 10^−1^
(1Z)‐1‐Propen‐1‐ylbenzene (α‐Methylstyrene)	117 + 118	9.19	MS	−92.78 (34.05)	−1.03 (0.51)	1.84 × 10^−3^	3.51 × 10^−3^
2‐Pentylfuran^a^	81 + 138	9.36	Std	3.06 (16.27)	0.05 (0.48)	6.73 × 10^−1^	6.95 × 10^−1^
3,7,7‐Trimethylcyclohept‐3‐ene (2‐Carene)	93 + 121	9.49	MS	−49.76 (25.52)	−0.56 (0.49)	1.92 × 10^−3^	3.51 × 10^−3^
2,2‐Dimethylpropionic acid butyl ester	57 + 103	9.65	MS	73.67 (15.18)	0.93 (0.51)	1.47 × 10^−4^	5.71 × 10^−4^
6‐Methyl‐5‐hepten‐2‐ol	70 + 95	9.94	MS	15.11 (11.08)	0.32 (0.49)	2.49 × 10^−1^	2.75 × 10^−1^
1‐Methyl‐4‐(1‐methylethenyl)‐cyclohexene (Limonene)	67	10.47	Std	−31.40 (22.20)	−0.40 (0.49)	1.54 × 10^−1^	1.84 × 10^−1^
1,2,3,4‐Tetrahydro‐1,5,7‐trimethylnaphthalene^a^	131 + 159 + 174	15.85	MS	−12.79 (17.64)	−0.19 (0.48)	9.86 × 10^−2^	1.27 × 10^−1^
1‐(2‐Methylphenyl)‐2‐propen‐1‐one	120 + 146	15.94	MS	−30.65 (29.25)	−0.29 (0.48)	2.61 × 10^−1^	2.80 × 10^−1^
1,1,6‐Trimethyl‐1,2‐dihydronaphthalene (TDN)^a^	157 + 172	19.76	MS	−61.68 (17.87)	−1.07 (0.52)	3.95 × 10^−5^	2.04 × 10^−4^
2‐Methoxy‐4‐prop‐2‐enylphenol (Eugenol)	149 + 164	19.85	Std	−12.49 (29.59)	−0.11 (0.48)	1.18 × 10^−1^	1.46 × 10^−1^
(E)‐1‐(2,3,6‐Trimethylphenyl)buta‐1,3‐diene	157 + 172	21.10	MS	−50.23 (14.49)	−1.02 (0.51)	1.20 × 10^−4^	5.32 × 10^−4^
1,1,5,6‐Tetramethyl‐1,2‐dihydronaphthalene	157 + 172	23.32	MS	−54.77 (34.65)	−0.47 (0.49)	3.68 × 10^−4^	9.51 × 10^−4^
2,5,8‐Trimethyl‐1,2,3,4‐tetrahydronaphthalen‐1‐ol	157 + 172	25.23	MS	−65.03 (35.54)	−0.57 (0.49)	1.66 × 10^−5^	1.03 × 10^−4^
[(2E,4E,6E,8E)‐3,7‐dimethyl‐9‐(2,6,6‐trimethylcyclohexen‐1‐yl)nona‐2,4,6,8‐tetraenyl] acetate (Retinol acetate)	119 + 268	38.91	MS	11.08 (50.31)	0.05 (0.48)	7.03 × 10^−8^	2.18 × 10^−6^
[(3S,8R,9S,10R,13S,14S)‐10,13‐Dimethyl‐17‐oxo‐1,2,3,4,7,8,9,11,12,14,15,16‐dodecahydrocyclopenta[a]phenanthren‐3‐yl] hydrogen sulphate (DHEA‐S)	91 + 270	39.43	MS	31.95 (22.56)	0.28 (0.48)	1.06 × 10^−6^	1.64 × 10^−5^
Unidentified VOCs							
VOC_1_	41 + 59	1.76	–	30.40 (12.24)	0.56 (0.49)	3.47 × 10^−2^	5.00 × 10^−2^
VOC_2_ a	145 + 163	20.86	–	−59.08 (16.15)	−1.13 (0.52)	5.15 × 10^−6^	3.99 × 10^−5^
VOC_3_	89 + 116 + 151	24.10	–	−21.54 (22.40)	−0.26 (0.48)	4.99 × 10^−2^	6.73 × 10^−2^
VOC_4_	157 + 172	24.15	–	−56.99 (24.51)	−0.70 (0.50)	9.05 × 10^−3^	1.48 × 10^−2^
VOC_5_	157 + 172	24.63	–	−70.87 (34.67)	−0.67 (0.50)	2.62 × 10^−4^	7.38 × 10^−4^
VOC_6_	83 + 132	26.85	–	−63.54 (24.97)	−0.80 (0.50)	1.08 × 10^−3^	2.39 × 10^−3^

The identification is proposed based on the NIST 14 (MS) and, when available, on the comparison with standards (Std). The percentage of variation (± % uncertainty), ES, ES_SE_, *P*‐values and *P*‐values corrected (*after BH‐FDR correction) are presented for each VOC.

a May have contribution from higher mean age of controls. The significant compounds after BH‐FDR correction are highlighted in light grey.

**Figure 2:**
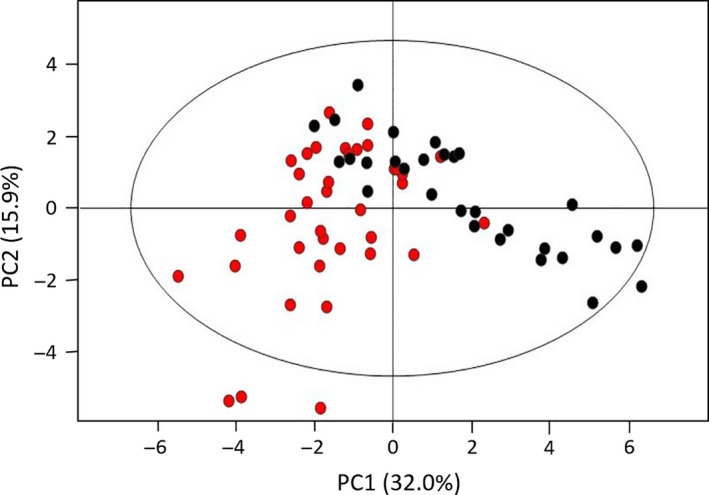
PCA scores scatter plot obtained for the urine of the unmatched cohort of controls (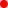) and RCC patients (●) with the 21‐metabolite panel, obtained with 2 PCs (ellipse indicates the 95% confidence level).

From the 21 compounds found significantly altered between the two classes of samples, 11 were found elevated in cancer patients compared to controls and 10 decreased. This 21‐panel of compounds includes five unidentified features (RT 20.86, 24.15, 24.63 and 26.85 min.). Using the normalized integrals of these 21 compounds, a PLS‐DA model was computed showing, after MCCV, a comparable performance regarding the sensitivity, specificity and CR but a relatively lower predictive power (*Q* ^2^) of the PLS‐DA model using all the selected variables for the unmatched cohort (median *Q* ^2^ = 0.46, sens = 90.8%, spec = 93.3%, CR = 92.1% *versus* median *Q* ^2^ = 0.72, sens = 95.6%, spec = 92.6%, CR = 93.9%; Table S3). Nevertheless, during unsupervised multivariate analysis (using a PCA), this 21‐compound panel was able to discriminate RCC and control classes, confirming the potential for them as RCC biomarkers (Fig. 2). Moreover, removing the VOC_2_ and 1,1,6‐trimethyl‐1,2‐dihydronaphthalene, that show possible bias with age, and resubmitting the remaining 19 normalized integrals to PCA analysis, the resulting model did not improve (PC1 = 32.8% and PC2 = 12.6% compared to PC1 = 32.0% and PC2 = 15.9%) and a similar result was observed for the PLS‐DA analysis (Table S3).

**Figure 3:**
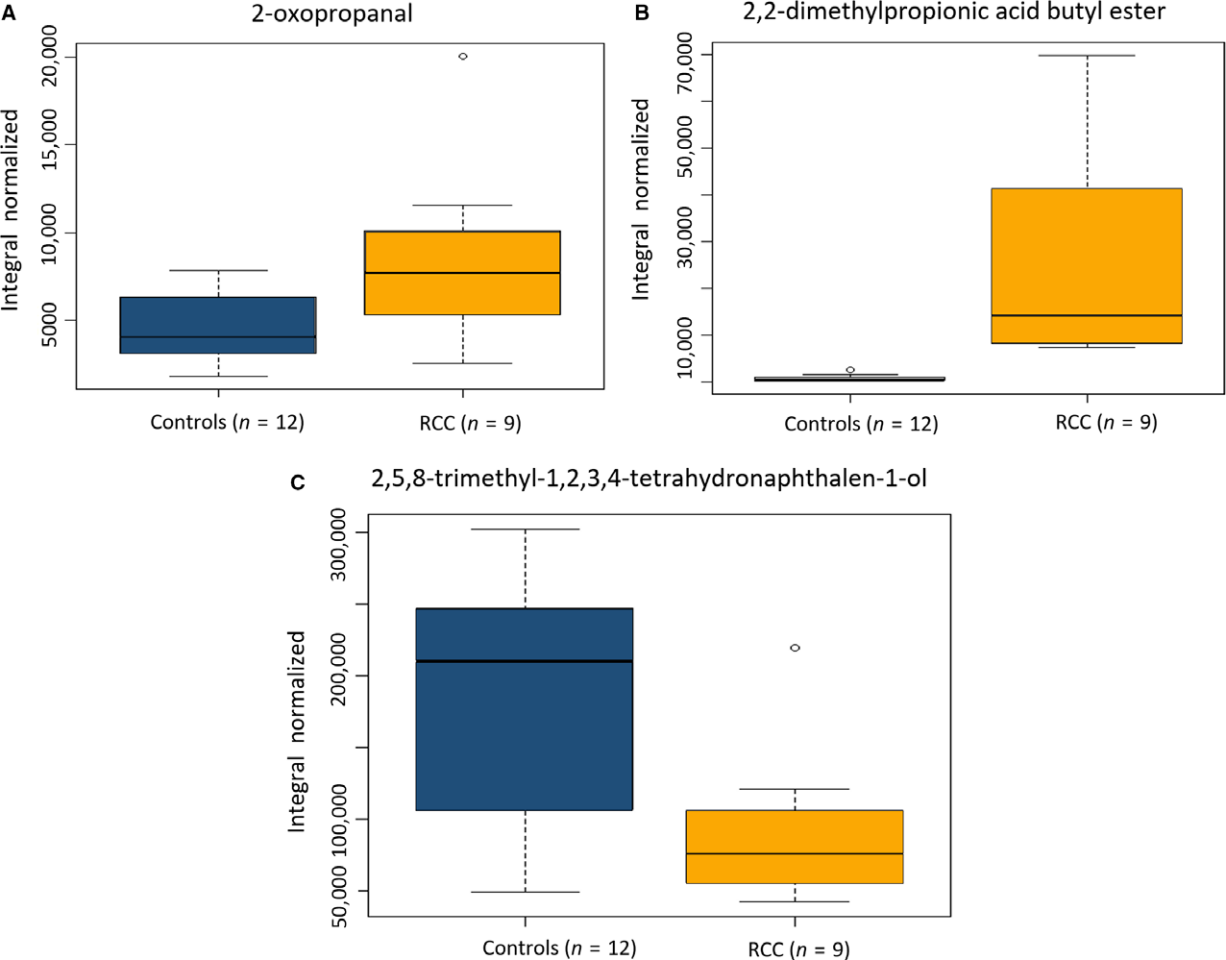
Boxplots for the three VOCs found significantly altered between urine controls (*n *=* *12) and RCC patients (*n *=* *9) samples in the independent set and that are in accordance with the results for the untargeted analysis. (A) 2‐oxopropanal (*P*‐value = 1.48 × 10^−2^), (B) 2,2‐dimethylpropionic acid butyl, (C) 2,5,8‐trimethyl‐1,2,3,4‐tetrahydronaphthalen‐1‐ol.

The significance of the 21 VOCs panel was further tested in an independent set of urine samples from 12 controls and nine RCC patients (Table S1). These samples were analysed under the same conditions but at a different time which led to significant visual differences on the *full scan* chromatograms compared to the typical chromatogram of the set used during the untargeted study (Table 1). In fact, from the 21 panel, only 15 were detected and only three of them showed to be statistically significant when comparing the RCC with controls: 2‐oxopropanal, 2,2‐dimethylpropionic acid butyl ester and 2,5,8‐trimethyl‐1,2,3,4‐tetrahydronaphthalene‐1‐ol. 2‐Oxopropanal and 2,2‐dimethylpropionic acid butyl ester were detected significantly increased in RCC urine samples compared to controls, whereas 2,5,8‐trimethyl‐1,2,3,4‐tetrahydronaphthalen‐1‐ol was decreased (Fig. 3), similar to what was found in the cohort of samples used for the untargeted approach, as seen in Table 2.

Additionally, the biomarker potential for these 21 compounds was tested in a small number of RCC samples with DMT2 to evaluate their sensitivity for the detection of RCC even in the presence of other comorbidities, particularly diabetes, which has been considered one of the potential risk factors for the development of RCC 27. From the 21‐panel, only five VOCs appeared statistically significant (*P* < 0.05) when comparing these RCC‐DMT2 samples with controls: 2‐oxopropanal, 2,2,5,5‐tetramethyltetrahydrofuran, α‐methylstyrene, 2,5,8‐trimethyl‐1,2,3,4‐tetrahydronaphthalen‐1‐ol, and DHEA‐S. Interestingly and confirming the potential of these compounds to detect RCC, the variation for all of them between RCC and controls was the same found during the untargeted study (Table 3).

**Table 3:** List of statistically significant varying metabolites in RCC patients with DMT2 (*n* = 7) compared to controls (*n* = 37), characterized by their IUPAC (and common) name. The percentage of variation (± % uncertainty), ES, ES_SE_ and *P*‐values are presented

Metabolite	% variation (± % uncertainty)	ES (±ES_SE_)	*P*‐value
2‐Oxopropanal	56.73 (20.10)	1.37 (0.86)	3.52 × 10^−2^
2,2,5,5‐tetramethyltetrahydrofuran	390.67 (76.91)	2.95 (1.02)	6.91 × 10^−5^
(1Z)‐1‐Propen‐1‐ylbenzene (α‐methylstyrene)	−94.41 (24.93)	−0.79 (0.83)	4.88 × 10^−2^
2,5,8‐Trimethyl‐1,2,3,4‐tetrahydronaphthalen‐1‐ol	−59.01 (23.11)	−0.51 (0.82)	8.94 × 10^−3^
[(3S,8R,9S,10R,13S,14S)‐10,13‐Dimethyl‐17‐oxo‐1,2,3,4,7,8,9,11,12,14,15,16‐dodecahydrocyclopenta[a]phenanthren‐3‐yl] hydrogen sulphate (DHEA‐S)	30.21 (25.11)	0.21 (0.81)	1.06 × 10^−3^

**Figure 4:**
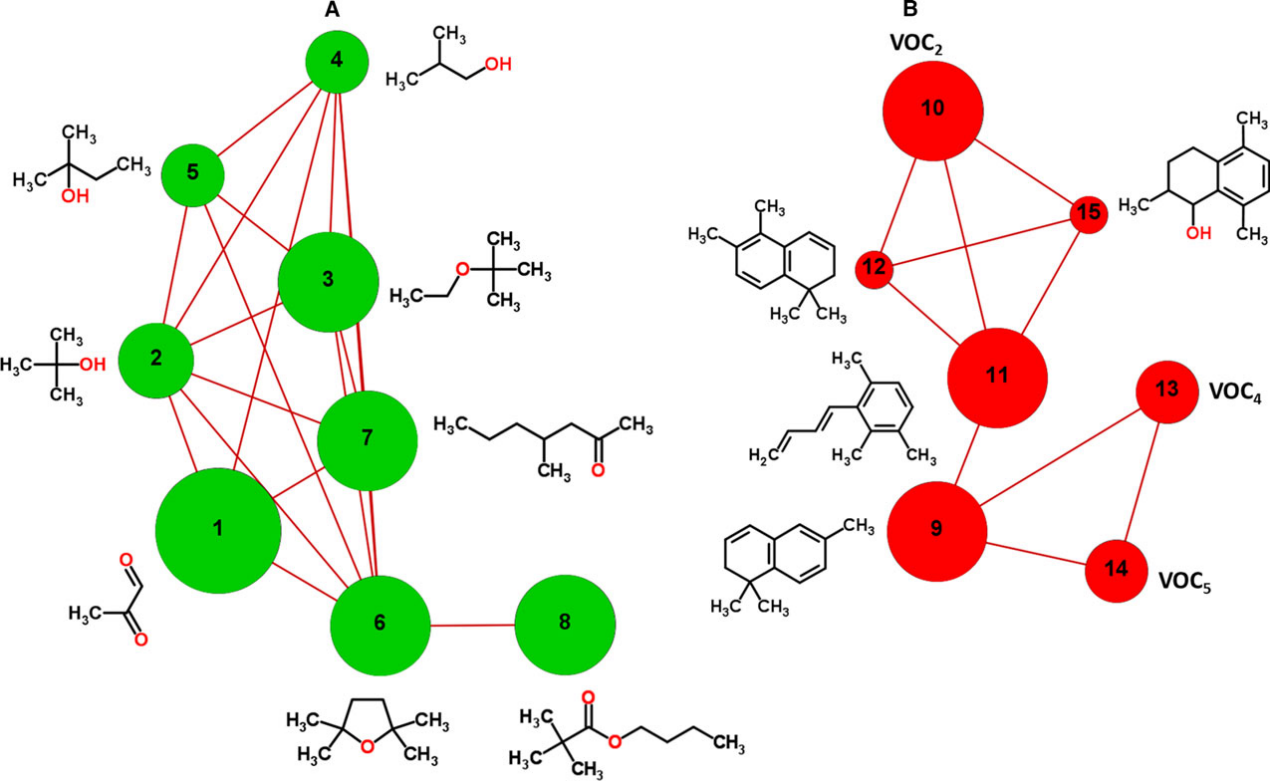
Correlation network of VOCs signature of RCC patients compared to controls, based on Spearman's correlation coefficients (|*r*| ≥ 0.7 and *P* < 0.01). Node size denotes the effect size value of RCC patients in relation to controls, while colours indicate direction of effect size with (A) red for decrease and (B) green for increase. Only positive correlations were found according to the threshold (|*r*| ≥ 0.7 and *P* < 0.01), as indicated by dark red lines. (Identification: (1) 2‐oxopropanal; (2) 2‐methylpropan‐2‐ol; (3) 2‐ethoxy‐2‐methylpropane; (4) 2‐methylpropan‐1‐ol; (5) 2‐methylbutan‐2‐ol; (6) 2,2,5,5‐tetramethyltetrahydrofuran; (7) 4‐methylheptan‐2‐one; (8) 2,2‐dimethylpropionic acid butyl ester; (9) TDN; (10) VOC2; (11) (E)‐1‐(2,3,6‐trimethylphenyl)buta‐1,3‐diene; (12) 1,1,5,6‐tetramethyl‐1,2‐dihydronaphthalene; (13) VOC4; (14) VOC5; (15) 2,5,8‐trimethyl‐1,2,3,4‐tetrahydronaphthalen‐1‐ol.

Finally, correlation networks were used to represent the overall interconnected map of the 21 VOCs found statistically different in RCC patients compared with controls with the aim to deduce underlying biochemical pathways based on the observed correlations. Interestingly, the resulting correlation network (Fig. 4) showed two correlation clusters comprising VOCs increased (A) and decreased (B) in RCC patients compared to controls. Cluster A reflects alterations in alcohols (2‐methylpropan‐2‐ol, 2‐methylpropan‐1‐ol and 2‐methylbutan‐2‐ol), carbonyl compounds (2‐oxopropanal and 4‐methylheptan2‐one) and ethers (2‐ethoxy‐2‐methylpropane and 2,2,5,5‐tetramethyltetrahydrofuran). In addition, 2,2,5,5‐tetramethyltetrahydrofuran is positively correlated with an ester compound (2,2‐dimethylpropionic acid butyl ester). Cluster B links the naphthalene derivatives (TDN, 1,1,5,6‐tetramethyl‐1,2‐dihydronaphthalene and 2,5,8‐trimethyl‐1,2,3,4‐tetrahydronaphthalen‐1‐ol) with (E)‐1‐(2,3,6‐trimethylphenyl)buta‐1,3‐diene. Moreover, this correlation network suggests that VOC_4_ and VOC_5_ may also be naphthalene derivatives due to their positive correlations with TDN, as well as common characteristic fragments in MS spectra (157 + 172 in Table 2).

## Discussion

In the present study, the volatile profile of human urine was studied, in an attempt to reveal a VOC‐biomarker panel for non‐invasive detection of this cancer. Despite the potential of VOCs for the discrimination between cancer and control samples being currently acknowledged 22, 24, 36, 37, 38, concerning RCC, the potentialities of human urine volatilome for that purpose are scarcely exploited 26, 39.

Previous studies have shown that, besides the metabolic status of the individual 11, other factors, such as gender 28, age 40 and diet 41, 42, may have a substantial impact on the urinary volatilome, which increases the importance of a critical analysis of each candidate urinary biomarker yielded by the analysis of volatiles in urine. In fact, the alterations on the volatiles may be not only a function of the presence of the disease but also of contributions from many other less uncontrolled variables which would decrease their biomarker power even when strong disease associations are found. Considering the present study, the impact of age, gender, BMI, and smoking habits on the urine volatilome was studied and showed to be negligible. In fact, only when gender‐matched controls over 60 years and under 60 years were compared, nine compounds (six of them already reported in human urine according to the HMDB) were found as being expressed differently in the urine of older and younger, with six being significantly increased. Robinson and Robinson (40), reported higher urinary levels of several ketones (namely carvone) and furans (such as 2‐methyltetrahydrofuran, 2‐methylfuran and 3‐methylfuran) in the urine of younger males compared to older (mean age not referred) 40. The alteration found for carvone and 2‐pentylfuran (decreased in the younger group) is concordant with that found in the present study but not for the other 2 ketones (1‐(2,3,6‐trimethylphenyl)‐3‐buten‐2‐one and 4‐(2,6,6‐trimethyl‐1‐cyclohexa‐1,3‐dienyl)butan‐2‐one), which were found here significantly increased in the older group. Moreover, the description of the biological origin and association of the volatiles found significantly altered with ageing is very difficult as the majority of them possess also an exogenous origin (diet).

Regarding the possible bias resultant from the unmatching of control and RCC urine samples for age, only four compounds showed possible bias: 2‐pentylfuran, 1,2,3,4‐tetrahydro‐1,5,7‐trimethylnaphthalene, TDN and VOC_2_. Thus, for the cohort considered, the effect of the disease on the urinary volatilome was not hindered by the unmatching of control and RCC samples for these unwanted sources of variation.

Among the compounds found significantly altered in the urine of RCC patients compared to controls, three alcohols (2‐methylpropan‐2‐ol, isobutanol and 2‐methylbutan‐2‐ol) were found significantly increased in the urine of RCC patients compared with controls, showing strong positive correlations between them. No previous cancer association was found for these compounds, but increased urinary levels of isobutanol, similar to other aliphatic alcohols and ketones, were already related with diabetes 28, 29, 43, while others reported decreased urinary levels of isobutanol in patients with diabetes 30, 44.

Considering carbonyl compounds, the increased levels of 2‐oxopropanal (also known as pyruvaldehyde) and 4‐methylheptan‐2‐one in urine of RCC patients showed a positive correlation, suggesting a possible relationship in the RCC disturbed biochemical pathways. 2‐Oxopropanal is a reactive carbonyl compound produced endogenously by the metabolism of acetone and the catabolism of dihydroxyacetone phosphate, glyceraldehyde‐3‐phosphate and threonine 45, 46. This metabolite has been found significantly increased in both urine and serum of patients with diabetes 45 and neurodegenerative diseases 47. Regarding cancer, the accumulation of 2‐oxopropanal (and consequently increased urinary excretion) may be a consequence of high glycolytic rates characteristic of cancer 46. In addition, increased levels of oxidative stress frequently observed in oncological states 48, 49 may contribute for even more accumulation of 2‐oxopropanal due to increased demands for glutathione. In this study, the association found between 2‐oxopropanal and RCC suggests, even more, a possible connection between diabetes and insulin resistance and RCC, which has been described as a possible risk factor for the development of this type of cancer 27. Other carbonyl compounds, such as ketones, were previously detected in the urine of oncological patients (colorectal, lymphoma and leukaemia) compared to controls 37; however, the present study reports increased levels of ketones (penten‐2‐one and 4‐methylheptan‐2‐one) in the urine of cancer patients as previously reported for breast cancer 50. In addition, penten‐2‐one has been detected in the faeces of patients with infectious diseases 51.

The decreased levels of (E)‐1‐(2,3,6‐trimethylphenyl)buta‐1,3‐diene, naphthalene derivatives and unknown VOCs (VOC_2_, VOC_4_ and VOC_5_) in urine of RCC patients may have a possible common origin, as suggested by the correlation network. Some naphthalene derivatives 52, 53 and (E)‐1‐(2,3,6‐trimethylphenyl)buta‐1,3‐diene 54 have been reported as exogenous compounds related with diet. However, increased levels of naphthalene derivatives were previously reported in the urine of patients suffering from breast 50 and colorectal cancers, lymphoma and leukaemia compared to controls 37. The later study also suggests that naphthalene derivatives may be the degradation products of steroids 37.

Finally, considering the 21 VOCs signature of RCC patients, no correlations were found for α‐methylstyrene, 2‐carene, retinol acetate, DHEA‐S and VOC_6_. The presence of 2‐carene in human urine was already described 55, and an isomer (4‐carene) was found increased in the urine of patients with breast cancer 50, whereas here a decrease was observed for RCC patients compared to controls. Retinol acetate (a derivative of vitamin A) possess an exogenous origin and have been also related with diet 56. DHEA‐S is a steroid hormone produced endogenously related with ageing in men but not clearly defined in women 57. It was found significantly decreased in the serum of patients with lung cancer 58 and decreased in the urine of patients with epithelial ovarian cancer 59, alterations consistent with those found in this study.

Our findings are different from the results of another unique similar study described until now in the literature 39 which detected statistically significant differences in 14 VOCs in RCC patients compared to controls. These differences could at least in part be dependent on differences in samples’ preparation (we used acidified urine samples) and the extraction procedure used, as the type of fibre.

Considering a second set of independent samples (from the same geographic origin), analysed under the same conditions but approximately 1 year before, from the 21 compounds, only 15 were detected and 3 (2‐oxopropanal, 2,2‐dimethylpropionic acid butyl ester, and 2,5,8‐trimethyl‐1,2,3,4‐tetrahydronaphthalen‐1‐ol) were significant and in agreement with the results from the untargeted approach. Moreover, alterations in VOCs have been strongly associated with diabetes 29, 30, 43, and as aforementioned, diabetes has been positively associated with the development of RCC 27, 60. For that reason, and as a preliminary study to validate the 21‐panel of VOCs found here to be descriptive of RCC, seven samples of RCC patients with DMT2 were tested for the significance of those compounds. Five of the compounds (2‐oxopropanal, 2,2,5,5‐tetramethyltetrahydrofuran, α‐methylstyrene, and 2,5,8‐trimethyl‐1,2,3,4‐tetrahydronaphthalen‐1‐ol, and DHEA‐S) were concordant with the results yielded by the untargeted approach. The lack of significance for all the other compounds may be a result of a confounding effect of diabetes on the urinary volatilome.

Among all the samples analysed, 2‐oxopropanal and 2,5,8‐trimethyl‐1,2,3,4‐tetrahydronaphthalen‐1‐ol (this only identified using NIST14 and RI) were consistently and concordantly expressed differently between controls and RCC. Thus, we can suggest that these are the volatile compounds that hold a greater biomarker power and robustness regarding the detection of RCC, particularly in order to be able to detect the disease in a population characterized by high intervariability as is the case of the human population. From these, 2‐oxopropanal, presenting preferentially an endogenous origin, has at least theoretically a greater biomarker strength and disease correlation being less affected by other uncontrolled variables.

In this study, the urinary volatilome did not allow the discrimination of RCC urine samples according to their histological subtype or even TNM staging. Thus, this seems to hamper the ability of urinary VOCs to distinguish RCC of lower risk or slower progression from those more aggressive. However, this may be a result of the limited cohort of samples that is not well representative of all the stages or histological subtypes, and it would be expected that considering a bigger cohort of samples, the detection of predictive or prognostic biomarkers would be possible. A following larger study is warranted to investigate and confirm this.

Regarding the biochemical interpretation of the differentially expressed RCC‐associated VOCs, it is required to take into account that some of them may not be directly cancer‐derived but be associated with other local or systemic body responses, such as inflammation and/or necrosis 11.

Despite the difficulties and discrepancies perceptible in published works, there is still a wish for the use of VOCs as disease biomarkers due to their relatively easy analysis and for that, it would be imperative to correctly identify and exclude exogenous compounds as well as to decrease the possibility of sample contamination from sample collection until analysis. Moreover, the simultaneous analysis of different biological samples could help to decrease the spectrum of unreliable biomarkers. Additionally, the understanding of VOCs origins as well as deeper knowledge about the pathophysiology of the disease should assist the identification of specific disease‐related VOCs.

## Concluding remarks

In the past years, distinctive volatile profiles have been associated with several pathophysiological processes what, along with their non‐invasive sampling nature, made them very attractive for disease's monitoring. In fact, there are some expressive cases of differences on volatiles underlying specific disease, such as the characteristic uremic breath odour of patients with chronic renal failure, the acetone‐like breath of diabetic patients with ketoacidosis and specific odours due to bacterial infections 61. The same was also reported regarding cancer which provided the sustained basis for the study of urinary volatilome as a path for the identification of diagnostic cancer biomarkers.

In the cohort of samples studied, in general, sources of unwanted variation, such as age, gender, BMI and smoking habits showed a minimal impact on the urine volatilome compared to the effect of the disease, not hampering its classification. A panel of 21 VOCs was identified in urine as successfully characterizing RCC. Moreover, only for some of them previous correlations with cancer were reported in urine. Regarding a primary way of external validation of this 21‐panel in independent urine samples, 2‐oxopropanal and 2,5,8‐trimethyl‐1,2,3,4‐tetrahydronaphthalen‐1‐ol were found significantly altered in the urine of RCC patients compared to healthy controls. This suggests the increased potential of them for the validation as RCC biomarkers. However, an independent validation of the complete volatile signature found here for RCC is much needed as a follow‐up of these results. Nevertheless, considering the fact that several metabolic alterations are characteristically shared by different types of cancer and the cancer in its full complexity comprehends a diverse group of relationships (cancer cell–cancer cell and cancer cell–host), it should be addressed in a holistic way. Thus, validation of a panel of biomarkers instead of single biomarkers should prevail and be followed to achieve the sensitivity and specificity required.

In conclusion, the results reported in the present study, despite some limitations, are very encouraging, confirming that the evaluation of the urinary volatile profile holds great potential regarding the diagnosis of RCC. Moreover, their biological and pathophysiological importance of these discriminatory VOCs is worthy of further exploration.

## Conflict of interest

The authors have no relevant affiliations or financial involvement with any organization or entity with a financial interest in or financial conflict with the subject matter or materials discussed in the manuscript apart from those disclosed. No writing assistance was utilized in the production of this manuscript.

## Research involving human participants

All procedures performed in studies involving human participants were in accordance with the ethical standards of the Ethics Committee of IPO‐Porto (CES76/2012) and with the 1964 Helsinki declaration and its later amendments or comparable ethical standards.

Informed consent was obtained from all individual participants included in the study.

## Supporting information


**Figure S1** Representative *full scan* chromatogram obtained for human urine.

**Figure S2** Q^2^ distributions (a and c) and ROC plots of true and permuted classes (b and d) obtained from the validation of the PLS‐DA models for the HS‐SPME/GC‐MS chromatograms of human urine of controls and RCC patients before (a and b) and after (c and d) the application of the variable selection's method.

**Table S1** List of urine samples collected for controls and RCC subjects, comprising number of samples, age and gender; and, for RCC patients, histopathological cancer type, TNM staging, presence or absence of metastases, smoking habits and BMI.
**Table S2** List of selected VOCs identified for the computed PLS‐DA models.
**Table S3** Results obtained by MCCV (500 iterations) of PLS‐DA models built for the disease (controls *vs* RCC patients); age and gender (controls only); BMI, smoking habits, RCC subtypes and stages (RCC patients only); and (* and **) for the disease models obtained using the 21 integrals and 19 integrals (no bias) found to vary with univariate statistical relevance (*P*‐value < 0.05).
**Table S4** List of varying metabolites in controls >60 years (*n* = 13) compared to controls ≤60 years (*n* = 12), characterized by their IUPAC (and common) name, RTs and quantifier ions (*m/z*).
