# Supplementary figures and images for: GC‐MS metabolomics‐based approach for the identification of a potential VOC‐biomarker panel in the urine of renal cell carcinoma patients
Source: J Cell Mol Med. 2017 Apr 4;21(9):2092–105. doi: 10.1111/jcmm.13132 (PMC5571542; doi:10.1111/jcmm.13132)

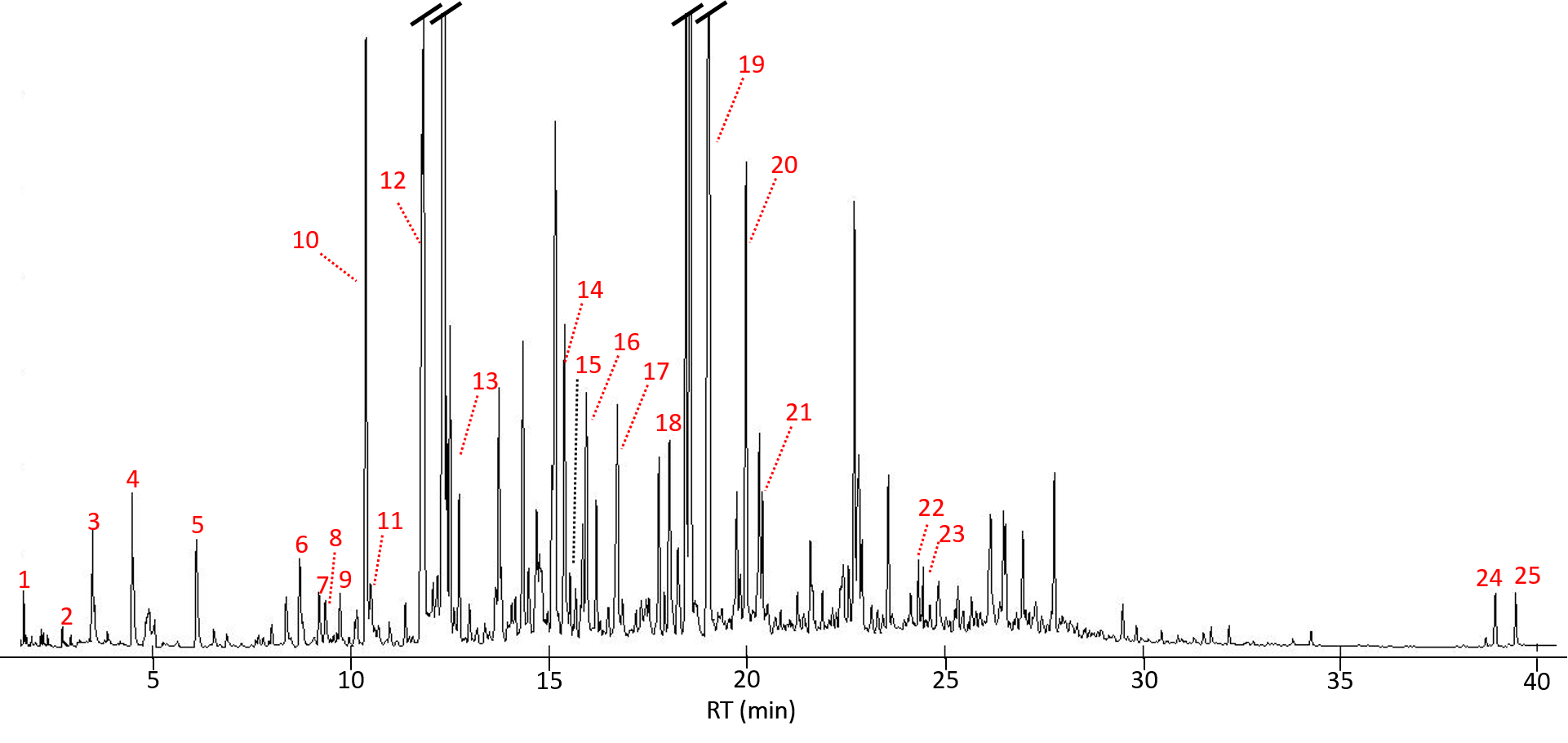

Supplement: Supplementary file 1 — Figure S1 Representative full scan chromatogram obtained for human urine. [file JCMM-21-2092-s001.tiff]

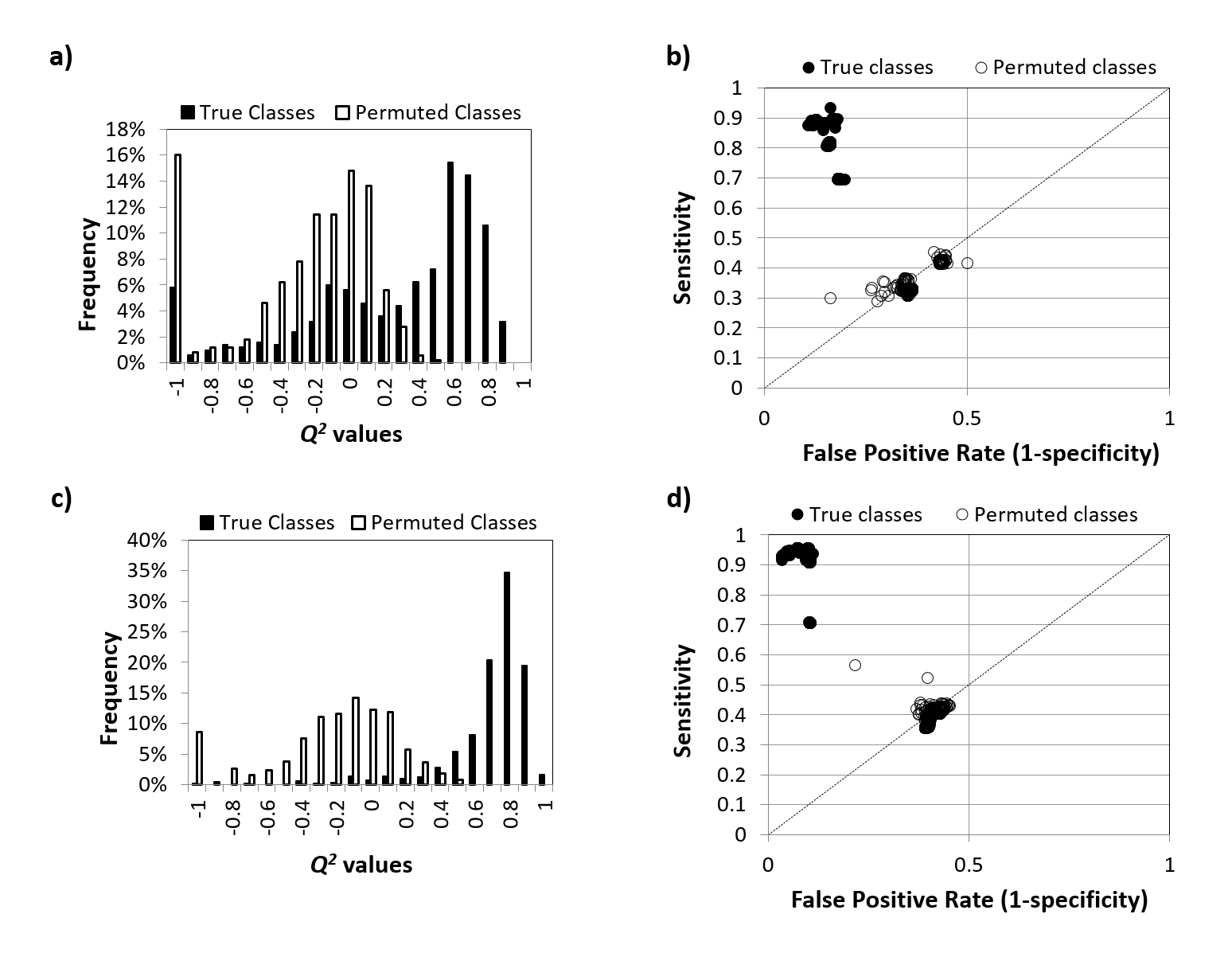

Supplement: Supplementary file 2 — Figure S2 Q2 distributions (a and c) and ROC plots of true and permuted classes (b and d) obtained from the validation of the PLS‐DA models for the HS‐SPME/GC‐MS chromatograms of human urine of controls and RCC patients before (a and b) and after (c and d) the application of the variable selection's method. [file JCMM-21-2092-s002.tiff]
