# Supplementary material for: GC‐MS metabolomics‐based approach for the identification of a potential VOC‐biomarker panel in the urine of renal cell carcinoma patients
Source: J Cell Mol Med. 2017 Apr 4;21(9):2092–105. doi: 10.1111/jcmm.13132 (PMC5571542; doi:10.1111/jcmm.13132)
Supplement: Supplementary file 3 — Table S1 List of urine samples collected for controls and RCC subjects, comprising number of samples, age and gender; and, for RCC patients, histopathological cancer type, TNM staging, presence or absence of metastases, smoking habits and BMI. Table S2 List of selected VOCs identified for the computed PLS‐DA models. Table S3 Results obtained by MCCV (500 iterations) of PLS‐DA models built for the disease (controls vs RCC patients); age and gender (controls only); BMI, smoking habits, RCC subtypes and stages (RCC patients only); and (* and **) for the disease models obtained using the 21 integrals and 19 integrals (no bias) found to vary with univariate statistical relevance (P‐value < 0.05). Table S4 List of varying metabolites in controls >60 years (n = 13) compared to controls ≤60 years (n = 12), characterized by their IUPAC (and common) name, RTs and quantifier ions (m/z). [file JCMM-21-2092-s003.docx]

**Supplementary information**

**Supplementary Table 1** List of urine samples collected for controls and RCC subjects, comprising number of samples, age and gender; and, for RCC patients, histopathological cancer type, TNM staging, presence or absence of metastases, smoking habits and BMI.^a^ Former smokers. ^b^ Includes 2 former smokers and 1 smoker. ^c^ Information not available for 1 subject

| **Sample group** | **n^o^. samples** | **Age range** | **Mean Age ± SD** | **Females** | **Males** |
| --- | --- | --- | --- | --- | --- |
| **Controls** | 12 | 38-83 | 59.08 ± 12.79 | 7 | 5 |
| **RCC** | 9 | 44-72 | 60.00 ± 8.76 | 6 | 3 |
| Clear- cell (ccRCC) | 4 | 53-72 | 63.75 ± 7.93 | 1 | 3 |
| Type 1 papillary (pRCC) | 1 | 53 | - | 1 | - |
| Chromophobe (chRCC) | 3 | 44-68 | 57.33 ± 12.22 | 3 | - |
| Unclassified | 1 | 60 | - | - | 1 |
| With metastases | 1 | 64 | - | - | 2 |
| Without metastases | 8 | 44-72 | 59.50 ± 9.23 | 6 | 2 |
| Stage I | 5 | 44-68 | 58.20 ± 9.86 | 4 | 1 |
| Stage II | 3 | 53-64 | 59.00 ± 5.57 | 2 | 1 |
| Stage III | 1 | 72 | - | - | 1 |
| Stage IV | - | - | - | - | - |
| Smokers ^a^ | 3 | 60-72 | 68.00 ± 5.66 | 1 | 2 |
| Non smokers | 6 | 44-68 | 57.71 ± 9.07 | 5 | 1 |
| BMI ≥ 25 | 5 | 53-72 | 62.40 ± 8.85 | 3 | 2 |
| BMI < 25 | 4 | 44-64 | 57.00 ± 8.87 | 3 | 1 |
| **RCC with DMT2** | 7 | 40-82 | 66.43 ± 14.13 | 3 | 4 |
| Clear- cell (ccRCC) | 4 | 40-73 | 64.00 ± 16.02 | 1 | 3 |
| Type 1 papillary (pRCC) | 2 | 55-82 | 68.50 ± 19.09 | - | 2 |
| Chromophobe (chRCC) | 1 | 72 | - | 1 | - |
| With metastases | 1 | 72 | - | 1 | - |
| Without metastases | 6 | 40-82 | 65.50 ± 15.24 | 5 | 1 |
| Stage I | 3 | 40-72 | 55.67 ± 16.01 | 1 | 2 |
| Stage II | 1 | 73 | - | - | 1 |
| Stage III | 1 | 71-82 | 75.00 ± 6.08 | 1 | 2 |
| Stage IV | - | - | - | - | - |
| Smokers ^b^ | 3 | 40-82 | 64.33 ± 21.78 | 0 | 3 |
| Non smokers | 4 | 55-73 | 68.00 ± 8.68 | 2 | 2 |
| BMI ≥ 25 ^c^ | 2 | 40-55 | 47.50 ± 10.61 | 0 | 2 |
| BMI < 25 | 4 | 71-82 | 74.25 ± 5.19 | 2 | 2 |

**Supplementary Table 2** List of selected VOCs identified for the computed PLS-DA models. For each compound is displayed the IUPAC (and common) name, RT, retention index calculated (RI_cal_) based on the *n*-alkanes series, average retention index according to literature (RI_lit_), identification method (ID method) with the forward and reverse % of match when the NIST14 was used, human metabolome database (HMDB) ID, CAS registry number, and reported presence in urine [[55](#_ENREF_55)] or other samples ([www.hmdb.ca](http://www.hmdb.ca)). NA: not available

| **Metabolite** | **RT (min)** | **RI_calc_** | **RI_lit_** | **ID method** | **HMDB ID** | **CAS** | **Samples** |
| --- | --- | --- | --- | --- | --- | --- | --- |
| 2-Oxopropanal (pyruvaldehyde) | 1.67 | - | - | Std | HMDB01167 | 78-98-8 | Urine, blood |
| 2-Methylpropan-2-ol | 1.82 | - | 491 | Std | HMDB31456 | 75-65-0 | NA |
| 2-Ethoxy-2-methylpropane | 2.17 | - | 615 | MS  (88.3/91.4) | NA | 637-92-3 | NA |
| 2-Methylpropan-1-ol (Isobutanol) | 2.23 | - | 625 | Std | HMDB06006 | 78-83-1 | Urine, feces |
| 2-Methylbutan-2-ol | 2.34 | - | 615 | MS  (88.1/88.9) | HMDB33772 | 75-85-4 | NA |
| Pentan-2-one | 2.74 | - | 685 | Std | HMDB34235 | 107-87-9 | Urine, feces, saliva |
| 2,2,5,5-Tetramethyltetrahydrofuran | 3.81 | - | - | MS  (72.6/86.9) | NA | 15045-43-9 | NA |
| 1-Methyl-1,4-cyclohexadiene | 3.86 | - | 790 | MS  (83.3/88.1) | NA | 4313-57-9 | NA |
| 4-Methylheptan-2-one | 7.80 | 943 | 943 | MS  (86.4/92.1) | NA | 6137-06-0 | NA |
| Phenol | 9.02 | 986 | 980 | Std | HMDB00228 | 108-95-2 | Urine, blood, feces, saliva |
| **(**1Z)-1-Propen-1-ylbenzene (α-methylstyrene) | 9.19 | 992 | 976 | MS  (85.1/86.3) | HMDB59899 | 98-83-9 | NA |
| 2-Pentylfuran | 9.36 | 998 | 993 | Std | HMDB13824 | 3777-69-3 | Urine, feces, saliva |
| 3,7,7-Trimethylbicyclohept-3-ene (2-Carene) | 9.49 | 1002 | 1001 | MS  (83.5/89.5) | HMDB59884 | 4497-92-1 | NA |
| 2,2-Dimethylpropionic acid butyl ester | 9.65 | 1008 | 999 | MS  (73.3/83.0) | NA | 5129-37-3 | NA |

| ***(Cont.)* Metabolite** | **RT (min)** | **RI_calc_** | **RI_lit_** | **ID method** | **HMDB ID** | **CAS** | **Samples** |
| --- | --- | --- | --- | --- | --- | --- | --- |
| 6-Methyl-5-hepten-2-ol | 9.94 | 1017 | 997 | MS  (65.8/70.6) | - | 1569-60-4 | NA |
| 1-Methyl-4-(1-methylethenyl)-cyclohexene (Limonene) | 10.47 | 1035 | 1030 | Std | HMDB03375 | 138-86-3 | Urine, blood, feces |
| 2-Methyl-6-methylene-7-octen-2-ol (Myrcenol) | 13.29 | 1128 | 1117 | MS  (83.7/86.2) | HMDB36107 | 543-39-5 | NA |
| 1,2,3,4-Tetrahydro-1,5,7-trimethylnaphthalene | 15.85 | 1220 | 1250 | MS  (86.6/89.3) | HMDB59696 | 21693-55-0 | Urine |
| 1-(2-Methylphenyl)-2-propen-1-one | 15.94 | 1224 | 1265 | MS  (65.5/69.6) | NA | 39627-60-6 | NA |
| (5S)-2-Methyl-5-prop-1-en-2-ylcyclohex-2-en-1-one  (D-carvone) | 16.75 | 1253 | 1246 | Std | HMDB04487 | 2244-16-8 | Urine |
| 3-Methyl-6-(1-methylethyl)-2-cyclohexen-1-one  (Piperitone) | 17.03 | 1263 | 1253 | MS  (79.9/86.1) | HMDB34975 | 89-91-6 | Urine, saliva |
| 1-H-Indole | 18.06 | 1300 | 1295 | MS  (82.5/89.5) | HMDB00738 | 120-72-9 | Urine, feces, saliva |
| 1,1,6-Trimethyl-1,2-naphthalene (TDN) | 19.76 | 1360 | 1354 | MS  (88.7/93.1) | HMDB40284 | 30364-38-6 | Urine |
| 2-Methoxy-4-prop-2-enylphenol (Eugenol) | 19.85 | 1363 | 1355 | Std | HMDB05809 | 97-53-0 | Blood, saliva |
| (E)-1-(2,3,6-Trimethylphenyl)buta-1,3-diene | 21.10 | 1417 | 1430 | MS  (82.2/89.6) | NA | NA | NA |
| 1-(2,3,6-Trimethylphenyl)-3-buten-2-one | 21.44 | 1431 | 1423 | MS  (72.1/73.5) | HMDB59697 | 54789-45-6 | Urine |
| 4-(2,6,6-trimethyl-1-cyclohexa-1,3-dienyl)butan-2-one | 21.73 | 1443 | 1424 | MS  (75.4/75.9) | HMDB37139 | 20483-36-7 | Urine |
| [(4Z)-4-(2,2-dimethyl-6-methylidene-cyclohexylidene)butan-2-ol](http://www.chemindustry.com/chemicals/026111886.html) | 22.37 | 1468 | 1466 | MS  (74.1/77.0) | NA | 68238-73-3 | NA |

| ***(Cont.)* Metabolite** | **RT (min)** | **RI_calc_** | **RI_lit_** | **ID method** | **HMDB ID** | **CAS** | **Samples** |
| --- | --- | --- | --- | --- | --- | --- | --- |
| 1,1,5,6-Tetramethyl-1,2-naphthalene | 23.32 | 1506 | 1529 | MS  (71.0/81.0) | NA | 220766-68-7 | NA |
| 1,1,4,5,6-Pentamethyl-2,3-dihydro-1H-indene | 23.52 | 1515 | 1523 | MS  (76.5/77.5) | NA | 16204-67-4 | NA |
| (1S)-4,7-dimethyl-1-propan-2-yl-1,2-dihydronaphthalene (α-Calacorene) | 24.54 | 1558 | 1542 | MS (78.3/89.8) | HMDB36453 | 21391-99-1 | Feces, saliva |
| 3-(3-tert-Butylphenyl)-2-methylpropanal | 25.04 | 1580 | 1543 | MS (73.5/74.9) | NA | 62518-65-4 | NA |
| 2,5,8-Trimethyl-1,2,3,4-tetrahydronaphthalen-1-ol | 25.23 | 1591 | 1611 | MS (73.9/79.0) | NA | 55591-08-7 | NA |
| Diphenylmethanone (Benzophenone) | 26.43 | 1639 | 1635 | MS  (84.4/86.2) | HMDB32049 | 119-61-9 | Urine,saliva |
| 2-[(E)-(2-Ethoxy-3,4-dimethyl-2-cyclohexen-1-yldene)methyl]furan | 27.78 | 1723 | 1723 | MS  (70.1/73.3) | NA | 55162-49-7 | NA |
| [(2E,4E,6E,8E)-3,7-Dimethyl-9-(2,6,6-trimethylcyclohexen-1-yl)nona-2,4,6,8-tetraenyl] acetate (Retinol acetate) | 38.91 | - | 2555 | MS  (71.1/71.3) | HMDB35185 | 127-47-9 | NA |
| [(3S,8R,9S,10R,13S,14S)-10,13-Dimethyl-17-oxo-1,2,3,4,7,8,9,11,12,14,15,16-dodecahydrocyclopenta[a]phenanthren-3-yl] hydrogen sulfate (DHEA-S) | 39.43 | - | 2635 | MS  (83.4/86.4) | HMDB01032 | - [651-48-9](http://www.sigmaaldrich.com/catalog/search?term=78590-17-7&interface=CAS%20No.&N=0&mode=partialmax&lang=pt&region=PT&focus=product) | Urine, blood |

|  |  |  |  |  |  |  |  |
| --- | --- | --- | --- | --- | --- | --- | --- |
|  |  |  |  |  |  |  |  |
|  |  |  |  |  |  |  |  |
|  |  |  |  |  |  |  |  |
|  |  |  |  |  |  |  |  |
|  |  |  |  |  |  |  |  |

Supplementary Table 3 Results obtained by MCCV (500 iterations) of PLS-DA models built for the disease (controls *vs* RCC patients); age and gender (controls only); BMI, smoking habits, RCC subtypes and stages (RCC patients only); and (* and **) for the disease models obtained using the 21 integrals and 19 integrals (no bias) found to vary with univariate statistical relevance (p-value < 0.05)

|  | **No variable selection** | | | | | **With variable selection** | | | |
| --- | --- | --- | --- | --- | --- | --- | --- | --- | --- |
|  | Median Q^2^ | | Sens (%) | Spec (%) | CR (%) | Median Q^2^ | Sens (%) | Spec (%) | CR (%) |
| **Control and RCC subjects** | | | | | | | | | |
| Controls (*n*=37) *vs* RCC (*n*=30) | | 0.42 | 69.7 | 81.7 | 76.3 | 0.72 | 95.6 | 92.6 | 93.9 |
| Controls (n=18) *vs* RCC (n=18)  (age- and gender-matched) | | 0.16 | 67.6 | 65.9 | 66.7 | 0.66 | 90.8 | 93.3 | 92.1 |
| Controls (n=13) *vs* ccRCC (n=13)  (age- and gender-matched) | | -0.18 | 70.3 | 69.9 | 70.1 | 0.18 | 82.3 | 82.1 | 82.2 |
| Controls (n=37) vs RCC (n=30) using 21 signal integrals* | | 0.46 | 96.8 | 80.8 | 87.9 |  |  |  |  |
| Controls (n=37) vs RCC (n=30) using 19 signal integrals** | | 0.35 | 96.6 | 83.6 | 89.4 |  |  |  |  |
| **Control subjects only** | | | | | | | | | |
| Age≤60 yrs (n=12) vs age>60 yrs (n=13) (gender-matched) | | -0.30 | 61.5 | 59.0 | 60.3 | 0.58 | 80.1 | 82.6 | 81.3 |
| Females (n=10) vs males (n=10)  (age-mathced) | | -0.75 | 61.0 | 38.6 | 50.9 | 0.04 | 83.5 | 78.2 | 81.1 |
| **RCC subjects only** | | | | | | | | | |
| Non-smokers (n=21) vs smokers (n=9) | | 0.04 | 49.7 | 77.0 | 68.5 | 0.06 | 35.2 | 88.4 | 71.9 |
| Non-smokers (n=9) vs smokers (n=9)  (age- and gender-matched) | | -1.33 | 84.3 | 53.0 | 74.9 | 0.20 | 81.2 | 77.8 | 79.5 |
| BMI<25 (n=7) vs. BMI≥25 (n=20) | | -0.50 | 79.1 | 8.6 | 62.7 | 0.36 | 96.0 | 30.0 | 80.6 |
| BMI<25 (n=7) vs. BMI≥25 (n=7) (age-matched) | | -0.14 | 15.0 | 60.0 | 37.5 | 0.40 | 79.3 | 67.9 | 73.6 |
| Other subtypes (n=9) vs. ccRCC (n=9) (age and gender-matched) | | -0.17 | 46.1 | 49.6 | 47.9 | 0.70 | 94.0 | 78.6 | 86.3 |
| Stages I+II (n=19) vs. Stage III and IV (n=11) (age- and gender-matched) | | -0.49 | 30.3 | 72.7 | 57.1 | 0.34 | 52.4 | 62.2 | 49.8 |
| Stage I (n=7) vs. Stage III and (n=7) (age- and gender-matched) | | -0.23 | 26.4 | 62.9 | 44.6 | 0.05 | 43.6 | 90.7 | 67.1 |

Supplementary Table 4 List of varying metabolites in controls > 60 years (n=13) compared to controls ≤ 60 years (n=12), characterized by their IUPAC (and common) name, RTs and quantifier ions (*m/z*). The identification is proposed based on the NIST 14 (MS) and, when available, on the comparison with standards (Std). The % of variation (± % uncertainty), ES, ES_SE_, *p*-values and *p*-values corrected (*after BH-FDR correction) are presented for each VOC. Only compounds statistically significant are presented

| **Metabolite** | **Quantifier ions (*m/z*)** | **RT (min)** | **Identification method** | **% variation**  **(± % uncertainty)** | **ES (±ES_SE_)** | ***p*-value** | **Corrected *p-*value** |
| --- | --- | --- | --- | --- | --- | --- | --- |
| 2-pentylfuran | 81+138 | 9.36 | Std | -58.15 (21.47) | -1.60 (0.90) | 7.00 X 10-5 | 1.96 X 10-3 |
| 1,2,3,4-tetrahydro-1,5,7-trimethylnaphthalene | 131+159+174 | 15.85 | MS | 92.60 (19.73) | 1.23 (0.86) | 6.07 X 10-3 | 2.83 X 10-2 |
| (5S)-2-methyl-5-prop-1-en-2-ylcyclohex-2-en-1-one (D-carvone) | 82+108 | 16.75 | Std | -95.58 (89.07) | -0.89 (0.82) | 4.51 X 10-3 | 2.83 X 10-2 |
| 1,1,6-trimethyl-1,2-dihydronaphthalene (TDN) | 157+172 | 19.76 | MS | 103.43 (26.08) | 1.01 (0.83) | 9.56 X 10-3 | 3.03 X 10-2 |
| VOC_2_ | 145+163 | 20.86 | - | 80.37 (24.54) | 0.91 (0.82) | 7.68 x10-3 | 3.03 x10-2 |
| 1-(2,3,6-Trimethylphenyl)-3-buten-2-one | 91+117+145 | 21.44 | MS | 98.68 (22.76) | 1.12 (0.84) | 9.75 x10-3 | 3.03 x 10-2 |
| 4-(2,6,6-trimethyl-1-cyclohexa-1,3-dienyl)butan-2-one | 177+192 | 21.73 | MS | 107.63 (22.93) | 1.17 (0.85) | 1.35 X 10-2 | 3.79 X 10-2 |
| (4Z)-4-(2,2-dimethyl-6-methylidene-cyclohexylidene)-butan-2-ol | 109+135 | 22.37 | MS | -75.50 (54.41) | -0.95 (0.83) | 5.08 X 10-3 | 2.83 X 10-2 |
| 1,1,4,5,6-Pentamethyl-2,3-dihydro-1H-indene | 173+189 | 23.52 | MS | 159.69 (20.32) | 1.67 (0.91) | 4.76 X 10-4 | 6.67 X 10-3 |

Supplementary Figure 1 Representative *full scan* chromatogram obtained for human urine. Legend: (1) 2-oxopropanal, (2) 2-pentenone, (3) dimethyl sulfide, (4) hexanal, (5) 4-heptanone, (6) dimethyl trisulfide, (7) phenol, (8) 2-pentylfuran, (9) octanal, (10) *o*-cymene, (11) E-2-nonenal, (12) *p*-cresol, (13) E-2-nonen-1-ol, (14) α-terpineol, (15) y-terpineol, (16) α-ionene, (17) carvone, (18) indole, (19) 4-vinylguaiacol, (20) eugenol, (21) β-damascenone, (22) 3,4-dehydro-β-ionone, (23) α-calacorene, (24) retinol acetate, (25) dehydroepiandrosterone sulfate (DHEA-S). Note: the common names for each compound are presented.

Supplementary Figure 2 Q^2^ distributions (a and c) and ROC plots of true and permuted classes (b and d) obtained from the validation of the PLS-DA models for the HS-SPME/GC-MS chromatograms of human urine of controls and RCC patients before (a and b) and after (c and d) the application of the variable selection’s method.
